# The Ontology Lookup Service, a lightweight cross-platform tool for controlled vocabulary queries

**DOI:** 10.1186/1471-2105-7-97

**Published:** 2006-02-28

**Authors:** Richard G Côté, Philip Jones, Rolf Apweiler, Henning Hermjakob

**Affiliations:** 1 European Bioinformatics Institute, Wellcome Trust Genome Campus, Hinxton, Cambridge, UK

## Abstract

**Background:**

With the vast amounts of biomedical data being generated by high-throughput analysis methods, controlled vocabularies and ontologies are becoming increasingly important to annotate units of information for ease of search and retrieval. Each scientific community tends to create its own locally available ontology. The interfaces to query these ontologies tend to vary from group to group. We saw the need for a centralized location to perform controlled vocabulary queries that would offer both a lightweight web-accessible user interface as well as a consistent, unified SOAP interface for automated queries.

**Results:**

The Ontology Lookup Service (OLS) was created to integrate publicly available biomedical ontologies into a single database. All modified ontologies are updated daily. A list of currently loaded ontologies is available online. The database can be queried to obtain information on a single term or to browse a complete ontology using AJAX. Auto-completion provides a user-friendly search mechanism. An AJAX-based ontology viewer is available to browse a complete ontology or subsets of it. A programmatic interface is available to query the webservice using SOAP. The service is described by a WSDL descriptor file available online. A sample Java client to connect to the webservice using SOAP is available for download from SourceForge. All OLS source code is publicly available under the open source Apache Licence.

**Conclusion:**

The OLS provides a user-friendly single entry point for publicly available ontologies in the Open Biomedical Ontology (OBO) format. It can be accessed interactively or programmatically at .

## Background

Controlled vocabularies and ontologies have evolved into essential tools in large-scale high-throughput scientific data annotation and retrieval. They ensure data consistency and increase the efficiency and accuracy of queries by standardizing the wide variations in terminology that may exist in a particular field of study. Although this variability might be understandable by humans, it can hamper systematic searches through large volumes of data (take for example the possible abbreviations, synonyms and acronyms for the yeast two hybrid experimental technique: Y2H, two-hybrid, 2H, etc). [1]

The Open Biomedical Ontologies project catalogues well-structured controlled vocabularies for shared use across different scientific domains [2]. To date, ontologies exist to describe the anatomy, developmental processes, phenotypes and pathologies of several species, as well as those oriented towards experimental and physical properties. For example, The Gene Ontology (GO), one of the oldest and richest ontologies, provides consistent descriptions of gene products in different databases in terms of their associated biological processes, cellular components and molecular functions in a species-independent manner [3,4]. The Medical Subject Headings (MeSH) thesaurus is another commonly used ontology produced by the National Library of Medicine and used for indexing, cataloguing, and searching for biomedical and health-related information and documents [5,6].

While such a plethora of information is available to the scientific community, the tools to make efficient use of it are less forthcoming. Individual projects provide code bases and database schemas that have controlled vocabulary sub-schemas where ontologies can be loaded (the chado schema from the Generic Model Organism Database (GMOD) project [7] or the Genomics Unified Schema (GUS) [8], for example). However, the ontology segment is only one part of a larger and more complex toolkit, possibly creating a larger overhead than required.

Each major ontology tends to have its own online browser (references 6, 9 and 10, among many others) yet there has been little effort to integrate these ontologies into a single point of query. One emerging project is the National Center for Biomedical Ontology, which will be responsible for maintaining the OBO library and creating biomedical data repositories and tools for accessing and using the data [11]. The Unified Medical Language System [12] is another initiative providing interactive and programmatic access to vocabularies, classifications and coding systems, though its focus is more oriented towards biomedical and clinical information sources and requires a licensing agreement and registration.

The second version of distributed annotation system protocol (DAS/2) [13,14] proposes ontology queries using a standardized URL scheme and XML responses. It will allow DAS clients to retrieve information about ontologies and terms and perform basic queries. However, the DAS/2 specification is still being drafted. Servers and clients that will implement it are still in development. One such server [15] currently only has 20 ontologies available and requires an understanding of the DAS protocol to use.

The BioMOBY project [16] is an interoperability system focusing on the integration of biological data and defines a protocol to link together distributed webservices to form workflows. It uses internal ontologies to explicitly define the data type and the relationships between them. Services are registered in a central repository that can be queried by users wishing to discover which services are available for specific data types. The BioMOBY ontologies are a means to define tool interoperability rather than being a data source. Ontology query services are provided by third parties who make them available via the MOBY Central registry [17]. However, the currently available services tend to be limited to either simple name queries, identifier queries or queries that return complex data types that are annotated with a given ontology term identifier. The services available are also restricted to a single ontology at a time (such as GO, EVOC or PO), generally the one being used by the party who provides the service.

There are to our knowledge no programmatic interfaces to allow for automated querying and interactive browsing of all OBO ontologies from a single interface.

Such interfaces would be useful in the creation of graphical user interface (GUI) widgets that could be integrated in the development of new tools and promote the use of ontologies in a simple yet powerful manner. Users would be more inclined to make use of controlled vocabulary terms if such data were available in applications used to generate, annotate or query scientific data.

## Implementation

### Overview

**Figure 1:**
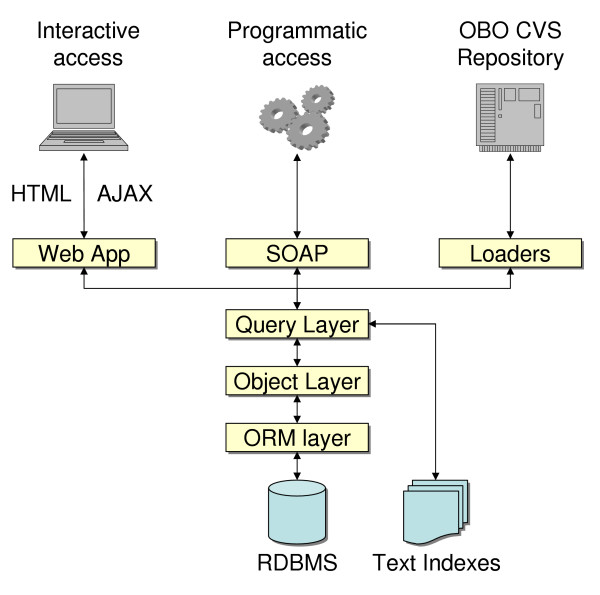
**OLS Architecture**. Loaders will connect to a CVS server to retrieve the latest versions of the ontology source files, transform the data into objects of the data model. The query layer is responsible for creating searchable text indexes as well as communicating with the database via the ORM layer. Programmatic access to the query layer is done via a SOAP interface. Interactive access is done via a Struts web application.

The Ontology Lookup Service (OLS) is a platform independent system that makes use of open source components and is written in Java. It is built around a core object model that is linked to a database using an object-relational mapping layer. Automated loaders are run on a daily basis to keep the ontologies up-to-date. Database queries can be performed interactively through a web application or programmatically through a SOAP interface [Figure 1].

### Data loading

The database model was inspired by the relevant portion of the BioSQL database schema. [18] Versions of the database schema currently exist for mySQL™ and Oracle™. Ontology loaders feed the database by parsing OBO-formatted flat-files and creating an object map that is persisted to the database using Apache ObjectRelationalBridge (OJB)[19]. All relevant information is extracted from the OBO file, including term accessions, names, synonyms, definitions, comments, relationships with other terms and cross-references with other ontologies and databases. The OLS does not do any curation on loaded ontologies, meaning that the data that is in the source flat-file is loaded faithfully. The OBO project maintains all of its ontologies in a CVS repository [2], making it easy to keep the database up-to-date. Updated files are obtained on a daily basis and any modified ontology will be loaded to the database. No loss of service is experienced during this process as the old version of the ontology is kept alive until the new one is fully loaded. Once loaded, the new version is set live and the old one is deleted.

Once the ontology has been persisted, another process will create an Apache Lucene [20] text index that will be used later on for case-insensitive full text queries. Terms are indexed on the preferred term name as well as on any annotated synonyms. Lucene has several advantages as a text-searching technology platform over RDBMS-based queries. It is very efficient at indexing and searching, it has a very powerful search syntax that can be used to limit and refine queries and it is platform independent, meaning that users do not need to rely on RDBMS-specific technologies to obtain good performance.

### Web application

**Figure 2:**
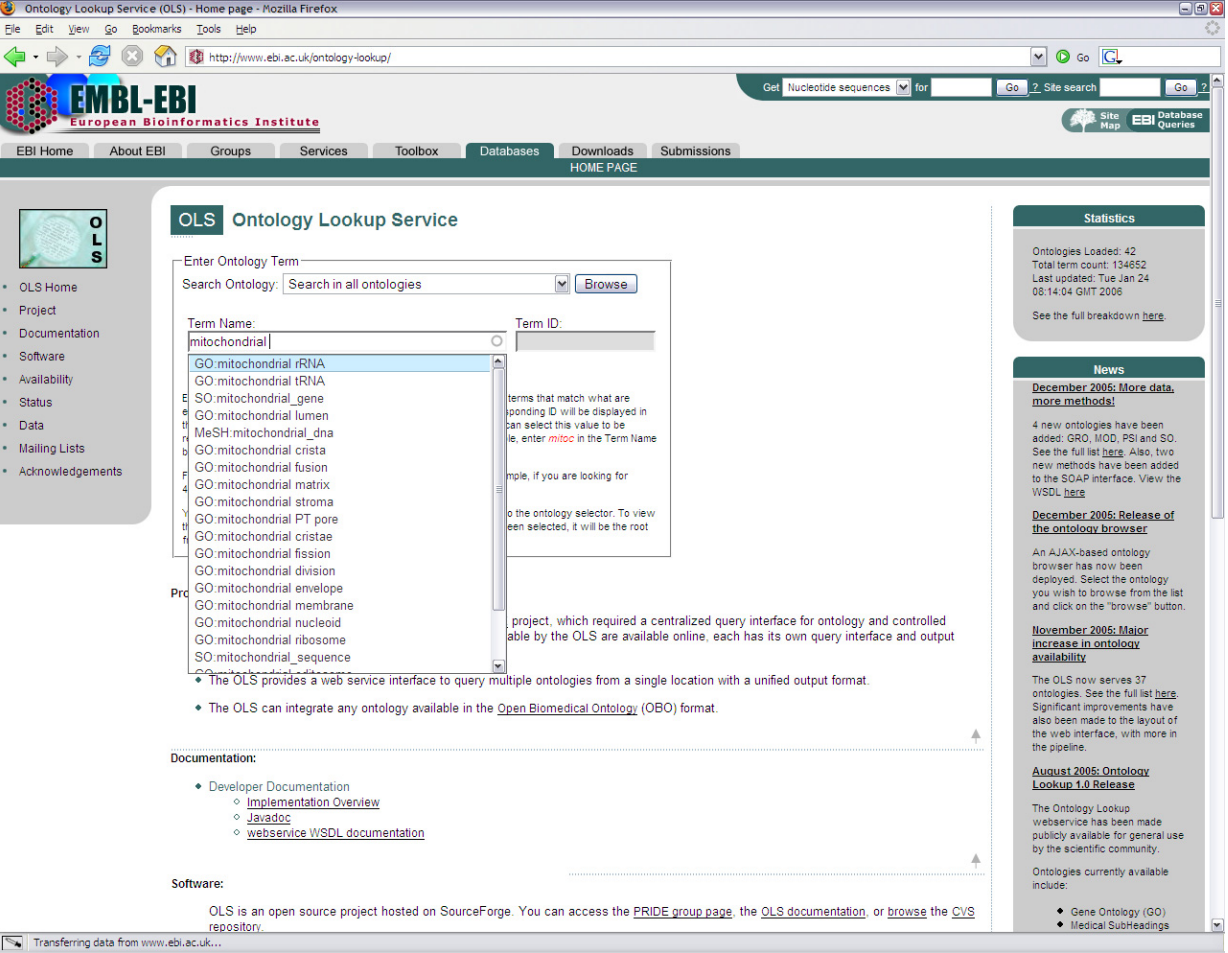
**AJAX-based auto-completion of a search term**. A list of possible suggestions will be updated as users type in a search term. If too many terms match the search keyword, users can select the "... and more" option at the bottom of the suggestion list (not shown in this figure). Users can search all ontologies at once or limit their search to a specific ontology.

**Figure 3:**
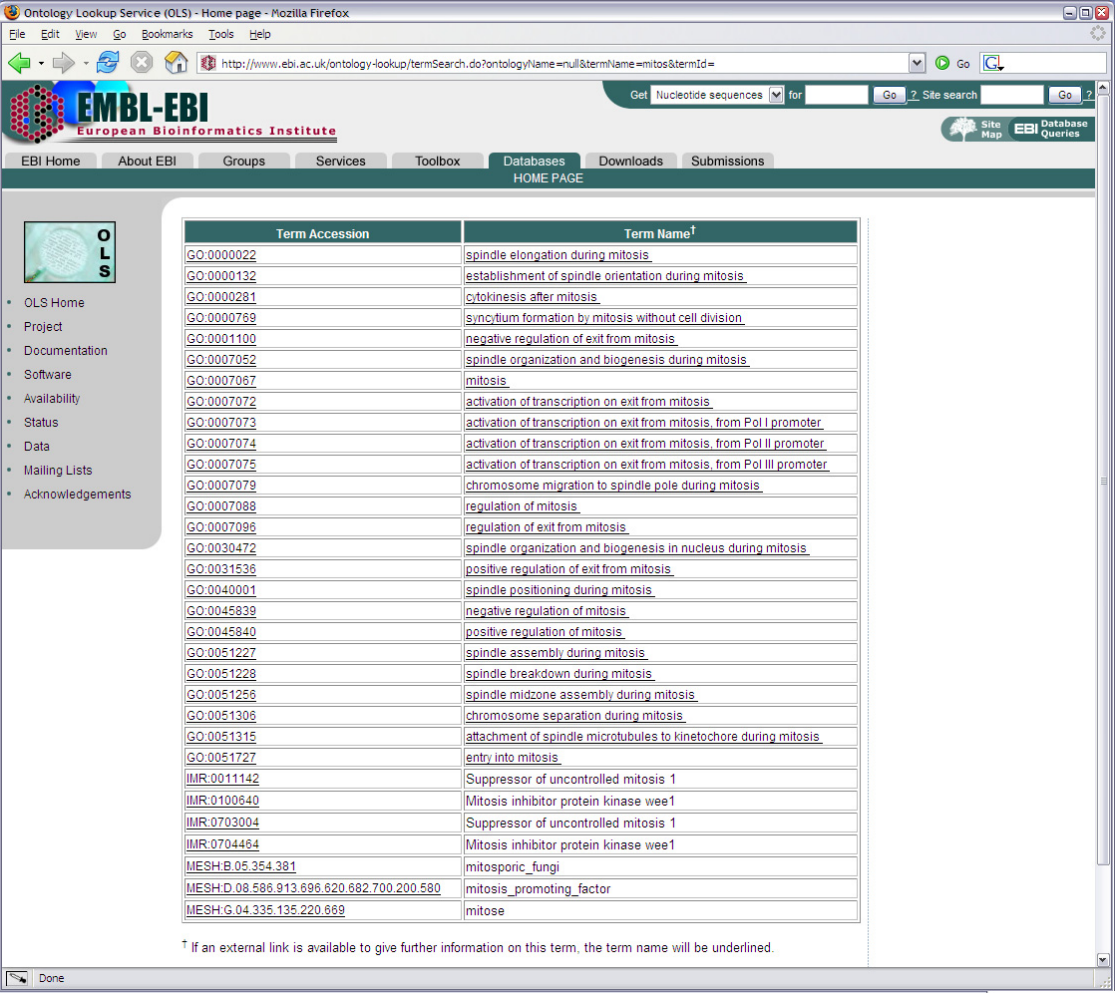
**Full listing of a search**. All the possible matches to a search keyword are listed in this page. Users can click on the term accession to return to the main page, which will display the definition and annotations stored for this term. When available, the user can click on the term name to be directed to term entry at the principal website associated with the selected ontology.

An interactive front-end was created using Java Server Pages (JSP) in the Struts Framework. From the OLS homepage, users can search for ontology terms using an auto-completing form. Users can select a specific ontology or search across all loaded ontologies. As users type a search term, a query is sent to a Java Servlet using Asynchronous JavaScript and XML (AJAX) once a search string is at least 3 characters long (excluding white spaces). A collection of close matches are sent back to the user, which are displayed in a drop-down menu [Figure 2]. Queries are done on the preferred term name as well as on any synonyms. If the exact term is in the list, the user can select it to obtain the preferred term accession id. Once a term is selected, a further AJAX request will return the definition for this term as well as any annotations associated with it (including definitions, comments and known synonyms). If the number of possible terms matching the search term exceeds a cut-off limit, the user has the possibility to see the full list by selecting the "... and more" option [Figure 3].

**Figure 4:**
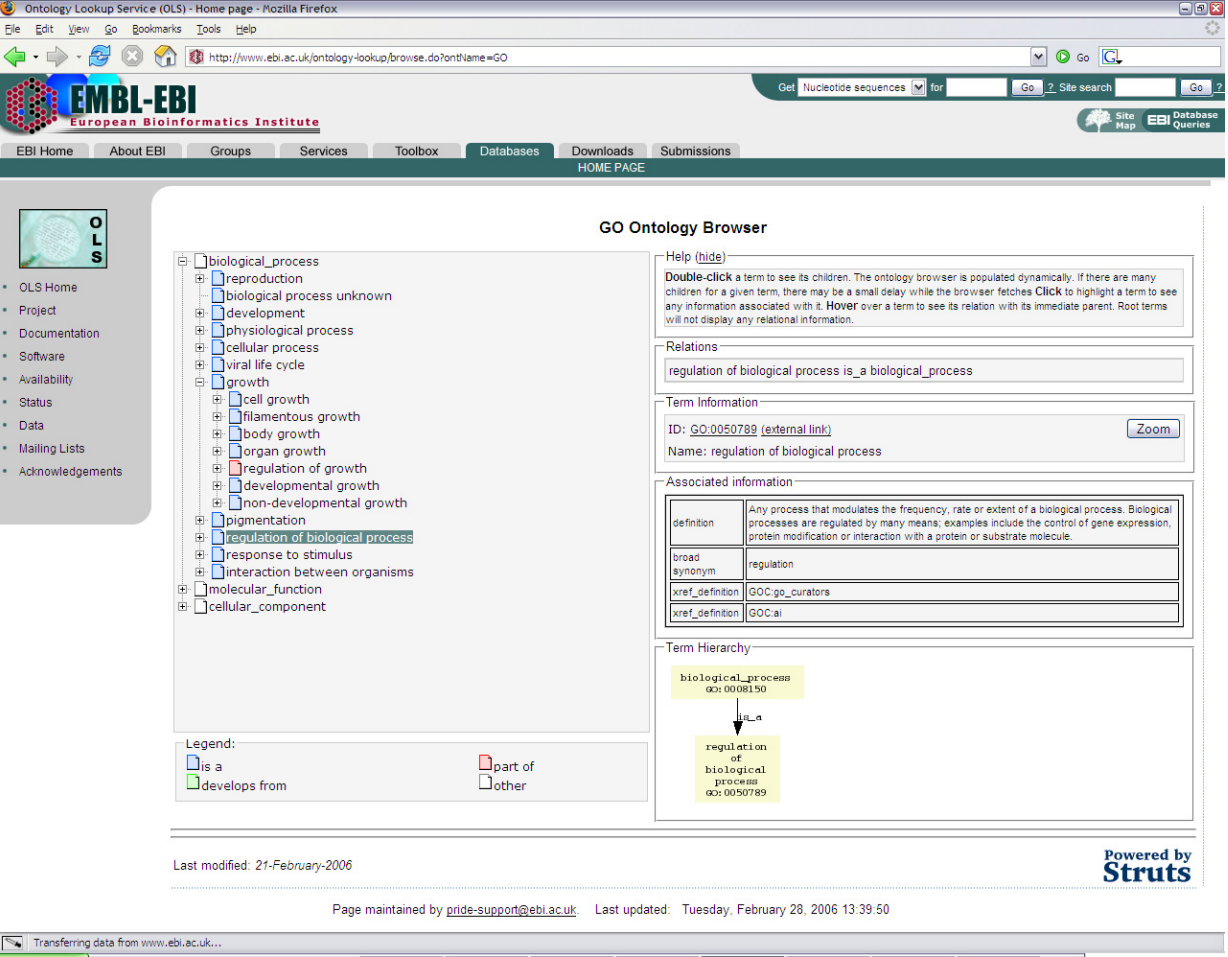
**AJAX-based ontology browser**. Users can browse a complete ontology or a subset of one by clicking on the ''browse'' button from the search form on the main page. The root term(s) of the ontology or subset are shown. Users can navigate the ontology dynamically by clicking on a term to load its children. Selecting a term will display the term name, accession, definition, synonyms and any annotations. Hovering over a term will display its relationship with its parent.

Users can also browse ontologies using a dynamically generated tree structure. Once an ontology is selected, the root terms of that ontology are displayed in the ontology browser. Clicking on a tree node will send an AJAX request to a Java Servlet which will return the child terms for this parent term and update the browser [Figure 4]. Selecting a term will display its definition and any annotations.

Relationships between terms are colour-coded to quickly provide an additional level of information. The three most significant relationships that comprise close to 98% of the relationships loaded in the OLS ("is a", 72%, "part of", 25% and "develops from", less than 1%) have been highlighted. Though several ontologies have defined custom relationship types, their usage is limited overall. To keep the interface simple, these relationships are colour-coded as "others" but hovering the mouse cursor over these terms will display the relationship type in the browser.

Users can also browse a subset of the ontology. This can be done by clicking on the "browse" button from the main page after a term has been selected from the auto-completion selections or by clicking on the "zoom" button from the ontology browser. This will re-root the browser on the selected term.

Although it would have been possible to generate a complete, fully-browsable tree for small ontologies, this would rapidly become cumbersome and inefficient for large ontologies such as GO, which have in excess of 20,000 terms. Using AJAX methodology, the tree is built up gradually as the user browses the ontology.

### SOAP service

Programmatic access to the database is available through a SOAP webservice. The webservice is implemented in Java and deployed using Apache AXIS [21]. Though the service makes internal use of the object model classes, only primitive data types are returned to help in platform interoperability. A server-side caching mechanism is implemented to store commonly accessed terms for increased performance. A sample java client connection class is made available to download from SourceForge [22]. The methods implemented in the webservice as well as detailed documentation of the webservice WSDL are available online at the OLS website. The OLS core API javadoc is also available online.

## Results

To date, 42 ontologies have been loaded into the OLS database, which account for close to 135,000 terms. A complete list of ontologies loaded into the OLS can be found online [23]. Currently, only ontologies available in the OBO flat-file format can be parsed into the OLS data model and persisted to the database. Future work will aim to create parsers for ontologies in the OWL format [24] as well as other controlled vocabularies of biological interest, such as the NEWT taxonomy [25].

Having a centralized point of query has proven to be useful for multiple projects at the EBI. This work started off as a requirement of the PRIDE project [26], which makes significant use of controlled vocabularies to annotate proteomic data sets [27]. Using AJAX to perform term auto-completion and definition lookups allows reusability of these components in other web applications. Since transmitted data volume is quite low, the speed at which the list of suggestions is refreshed will closely match the typing speed of most users. Work is currently underway to incorporate these widgets into the PRIDE and IntAct [28] web interfaces at the EBI.

The programmatic SOAP interface is already being used by the PRIDE project to query the ontologies and obtain constantly updated terms while importing and exporting datasets. Work is also underway to use the SOAP interface in annotation and curation tools to edit and maintain the data in PRIDE.

## Conclusion

The Ontology Lookup Service provides interactive and programmatic access to multiple ontologies, using lightweight and consistent interfaces. Users can perform simple queries using an interactive suggest-as-you-type form and browse ontologies in a clear tree-like browser. More sophisticated queries can be performed programmatically using a platform-independent SOAP interface. The service currently holds 42 ontologies covering fields such as anatomy, pathology, development, genomics, proteomics and experimental methods, among others. It is our hope that by providing generic, reusable code components, other projects in the bioinformatics community will make use of the ontology lookup service. Future work aims to increase the number of ontologies available to the general public and to enrich the SOAP interface from user feedback requirements. Users are encouraged to contact the authors to discuss feature requests to the interface. The data model contains more information than was required for the initial release requirements and could be made available if requested. Finally, many biomedical ontologies are available in OWL format and we hope to have OWL loaders for the next major release of the OLS.

## Availability and requirements

• **Project name: **Ontology Lookup Service

• **Project home page: **

• **Operating system(s): **Platform independent

• **Programming language: **Java

• **Other requirements: **Java 1.4, Tomcat 5.0, mySQL or Oracle

• **License: **Apache License 2.0

• **Any restrictions to use by non-academics: **none

## List of abbreviations

AJAX Asynchronous JavaScript and XML

CVS Concurrent Versioning System

DAS Distributed Annotation System

GO Gene Ontology

GMOD Generic Model Organism Database

GUI Graphical User Interface

GUS Genomics Unified Schema

MeSH Medical Subject Headings

OBO Open Biomedical Ontologies

OJB Object Relational Bridge

OLS Ontology Lookup Service

ORM Object Relational Mapping

OWL Web Ontology Language

PO Plant Ontology

RDBMS Relational Database Management System

RFC Request for Comments

SOAP Simple Object Access Protocol

XML Extensible Markup Language

## Authors' contributions

RC designed and implemented the data loading strategy, data indexing and querying as well as GUI component design and implementation. PJ participated in the design of the overall architecture and the design of the data model and ORM mapping. All authors read and approved the final manuscript.
